# A Novel Surgical Technique for the Reconstruction of Chronic Tennis Leg Injury

**DOI:** 10.7759/cureus.53943

**Published:** 2024-02-09

**Authors:** Nikolaos Platon Sachinis, Emmanouil Pantelidis, Byron Chalidis, Christos Koukos, Givissis Panagiotis

**Affiliations:** 1 First Orthopaedic Department of Aristotle University of Thessaloniki, "Georgios Papanikolaou" Hospital, Aristotle University of Thessaloniki, Thessaloniki, GRC

**Keywords:** operative treatment, chronic, rupture, medial gastrocnemius, tennis leg

## Abstract

Tennis leg, a rupture of the medial head of the gastrocnemius muscle at the musculotendinous junction (MTJ), is common, particularly among middle-aged sports enthusiasts. While acute cases usually resolve with conservative care, optimal surgical strategies for the treatment of chronic injuries remain undefined. This study reviews the current literature and details the successful operative treatment of a 37-year-old male with a 12-month history of tennis leg, employing a novel reverse flap technique from the MTJ's aponeurosis and augmented by a facia lata allograft.

## Introduction

Tennis leg, manifesting as a rupture of the musculotendinous junction (MTJ) of the medial head of the gastrocnemius muscle or, less commonly, the plantaris tendon, is a frequently encountered injury among active middle-aged individuals [1-3]. While the majority of acute cases respond favorably to conservative treatment modalities, leaving minimal long-term functional deficits [1,4,5], chronic manifestations of this injury are less common but often more complex and challenging to manage, frequently necessitating surgical intervention [6,7].

Despite the prevalence and recognition of this condition, the medical community has not reached a consensus regarding the most effective surgical approach for the treatment of chronic tennis leg injuries [6,7]. This report introduces a novel surgical technique aimed at addressing chronic tennis leg, drawing from the case of a 37-year-old male patient who experienced prolonged symptoms following an initial acute injury. The persistent nature of his condition, coupled with the findings upon physical examination, highlighted the limitations of conservative management and underscored the need for a more definitive surgical solution. Furthermore, this report includes a comprehensive review of the current literature on tennis leg injuries, examining various surgical techniques and treatment outcomes for such cases [4,8,9].

## Case presentation

A 37-year-old male presented to our outpatient department with a chronic complaint of pain in his right calf, persisting for the last 12 months. The patient reported an acute onset of pain during a recreational football game, characterized by a sudden 'tearing sensation' in the right calf, followed by immediate swelling and inability to bear weight on the affected limb. The initial diagnosis at the time of injury was a gastrocnemius strain, for which he received conservative treatment. Although the acute symptoms subsided over the following weeks, the patient reported persistent, less intense pain accompanied by weakness and rapid onset of soreness during physical activities, ultimately impacting his ability to participate in sports.

Physical examination revealed noticeable asymmetry between the two sides with a diminished muscular contour and a palpable, tender gap at the right medial gastrocnemius head. Ankle plantar flexion on the affected side exhibited mild to moderate weakness compared to the unaffected limb.

**Figure 1:**
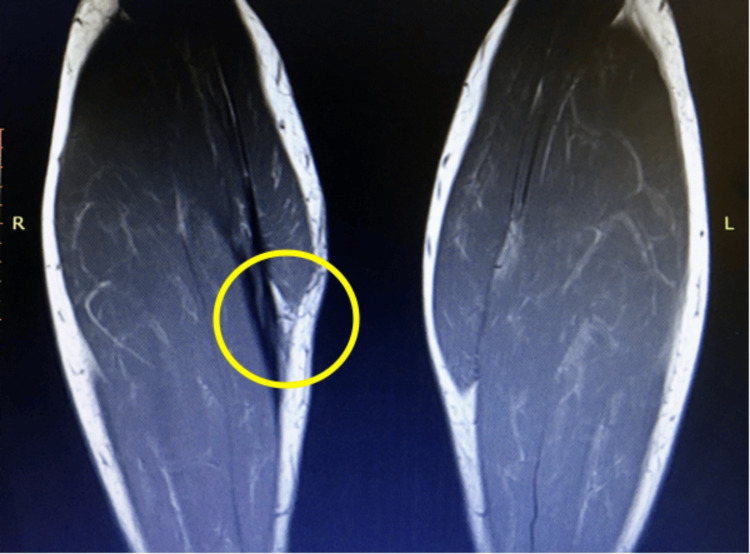
T2 weighted coronal image of both legs focusing on the gastrocnemius muscle. Scarring of the right medial gastrocnemius muscle can be seen as grey lines coming from the muscle head going peripherally to the tendon, and this has been highlighted with a yellow circle.

Subsequent soft tissue ultrasound examination was done immediately on site and it disclosed a full-thickness rupture of the medial gastrocnemius head at the level of the MTJ with an approximately 4 cm cephalic retraction of the muscle belly. Indications of chronicity, such as fatty degeneration and focal intramuscular calcifications, were also noted. To further delineate the extent and nature of the injury, an MRI was performed approximately a week later, revealing signs of a chronic condition, including moderate subcutaneous edema and the previously mentioned features (Figure 1).

**Figure 2:**
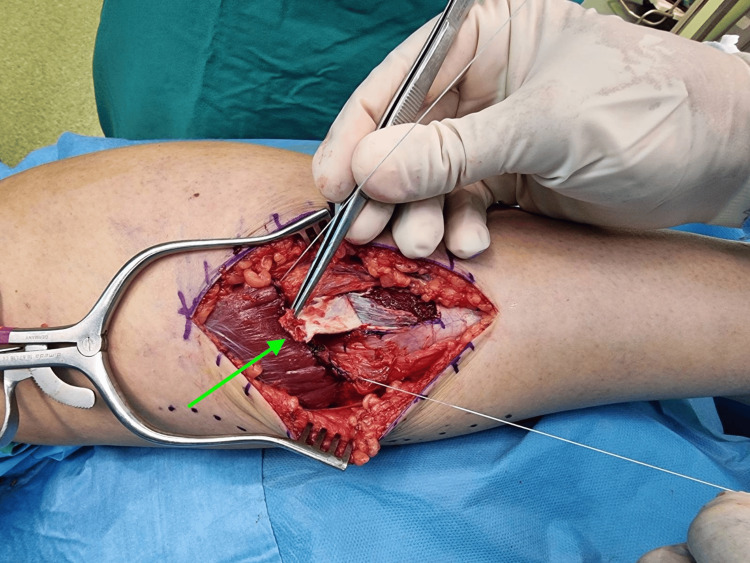
Intraoperative image of the technique employed to manage the chronic injury of the right medial gastrocnemius muscle. Scar removal and muscle attachment onto its aponeurosis. Reverse flap created (green arrow), and sutured to the muscle belly.

**Figure 3:**
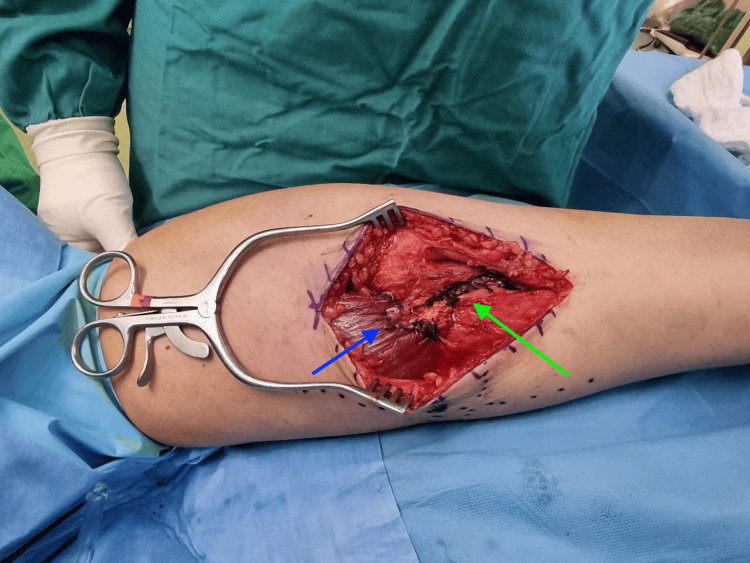
Intraoperative image of the technique employed to manage the chronic injury of the right medial gastrocnemius muscle. Reverse rectangular flap from the aponeurosis has been securely sutured onto the muscle belly (blue arrow); the rest aponeurosis has been also sutured in a side-by-side manner (green arrow).

**Figure 4:**
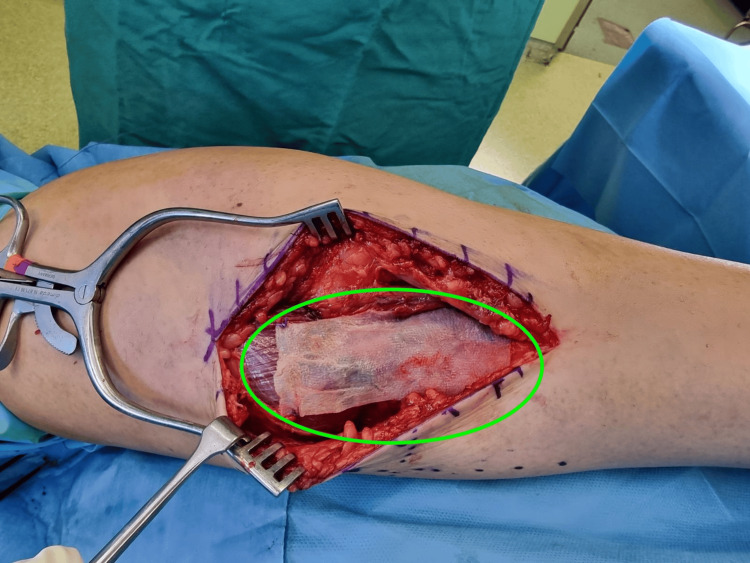
Intraoperative image of the technique employed to manage the chronic injury of the right medial gastrocnemius muscle. Fascia lata allograft being superimposed on the medial head and aponeurosis of the gastrocnemius (green circle) and readied to be sutured in place.

Given the chronicity of the injury and the persistence of the patient's symptoms, a decision was made to proceed with operative treatment. The patient was positioned prone under general anesthesia, and a careful dissection was performed to expose the ruptured MTJ of the medial gastrocnemius. Care was taken not to damage the sural nerve. A meticulous excision of the scar tissue was conducted, followed by repositioning and suturing of the medial gastrocnemius head onto its aponeurosis using absorbable sutures. A reverse rectangular flap was then created from the gastrocnemius aponeurosis and secured onto the muscle belly. To reinforce the repair, a fascia lata allograft was superimposed on the medial head and aponeurosis of the gastrocnemius and loosely sutured in place (Figures 2-4).

Postoperatively, the patient's leg was immobilized with a below-knee dorsal splint in the resting equinus for three weeks, followed by a full cast in a neutral position for an additional three weeks. The surgical wound healed without complications, and the patient was instructed to avoid weight-bearing on the right lower limb for the first six weeks postoperatively. Then, the cast was removed, and partial weight-bearing was allowed, gradually progressing to full weight-bearing by the eighth week. Range of motion exercises commenced after cast removal and by twelve weeks postoperatively the patient transitioned into muscle-strengthening resistance exercises.

**Figure 5:**
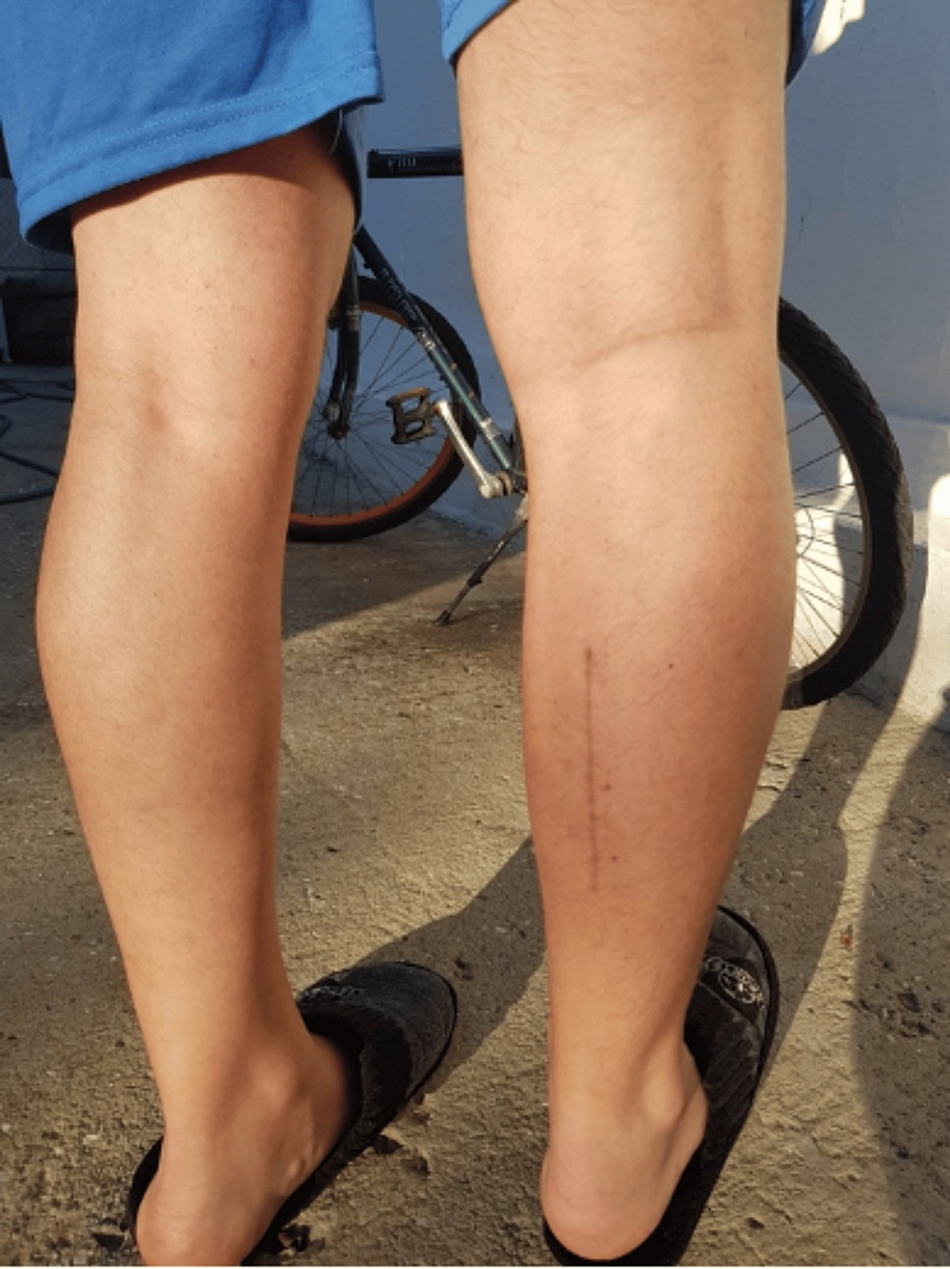
Final appearance of the patient’s leg and scar wound 12 months after the operation.

By the fourth month postoperatively, the patient regained full range of motion in plantar flexion and dorsal extension of the ankle, and by the sixth month, the strength of the right leg's plantar flexion was comparable to the left. The patient reported substantial improvement in functionality and was able to return to playing football without significant limitations. To quantify the patient's perception of the outcome, the Achilles Tendon Total Rupture Score (ATRS) was employed. The preoperative ATRS was 60, which significantly improved to 88, six months post-surgery, indicating a favorable patient-reported outcome. At 12 months it reached 98; the patient expressed overall satisfaction with the end result and no regrets undergoing this operation (Figure 5).

## Discussion

Tennis leg, historically characterized as the traumatic rupture of the plantaris tendon, is now better understood as typically involving a complete or partial tear of the musculotendinous junction (MTJ) of the medial head of the gastrocnemius muscle [1,8,10]. This condition predominantly affects active, middle-aged individuals and is precipitated by movements that overstress the calf muscles, such as plantar flexion or resisted passive dorsiflexion of the ankle with an extended knee [2,4].

The conventional treatment for acute instances of tennis leg is largely conservative, focusing on rest, ice, compression, and elevation (RICE), which is often successful [1,4]. However, chronic cases present a more complex clinical challenge, often necessitating surgical intervention, especially when conservative measures fail to resolve symptoms [6,7,11,12] (Table 1).

**Table 1:** Literature related to chronic leg injuries management.

Author(s)	Year	Study Type	Key Findings
Gaulrapp [11]	1999	Clinical study	Examined tennis leg using ultrasound and discussed a conservative therapy program based on grading the lesion.
Kwak et al. [4]	2006	Clinical Study	Explored outcomes of conservative treatment and compression effects.
Cheng et al. [6]	2012	Case Series	Discussed rare cases where surgical intervention is indicated for tennis leg, typically for longstanding ruptures.
Jennings & Peterson [7]	2013	Technical Note	Described a "trap door" surgical technique for repairing chronic injuries.
Ryu et al. [12]	2017	Case report	Reported severe concomitant contracture of the knee and ankle joint due to a maltreated gastrocnemius muscle rupture

Our review of the literature highlights several important studies relevant to the surgical treatment of chronic tennis leg: Kwak et al. and Cheng et al. discuss rare instances where surgical intervention becomes necessary, typically for long-standing ruptures. These studies underscore the potential necessity for surgery in chronic cases unresponsive to conservative treatment [4,6]. Jennings & Peterson describe a "trap door" surgical technique for repairing chronic injuries, offering a potential approach that could be compared with the novel technique we propose [7]. 

Ryu et al. report on severe complications due to a maltreated gastrocnemius muscle rupture, indicating that delayed or inadequate treatment can lead to conditions necessitating more complex surgical intervention [12]. Also, Gaulrapp discusses the importance of accurately diagnosing and grading the severity of the injury to inform treatment, underlining the role of conservative treatments and the need to understand when these might be insufficient [11].

In the context of our case, the literature underscores the nuanced decision-making process required when managing chronic tennis leg. While conservative treatments are often the first line of defense, the reviewed studies collectively suggest a pivot to surgical intervention in chronic or complicated cases. The "trap door" technique described by Jennings & Peterson [7], for example, is an innovative approach, yet our proposed reverse flap technique from the aponeurosis of the gastrocnemius MTJ with additional reinforcement by fascia lata allograft might offer a more robust solution for the degenerated nature of chronically ruptured tissues seen in cases like ours. 

Moreover, the complications highlighted by Ryu et al [12] demonstrate the potential severity of untreated or inadequately treated tennis leg, emphasizing the importance of timely and effective intervention. The study by Gaulrapp [11] further supports the need for a careful and detailed assessment of the injury's severity, which not only guides initial treatment but also informs the potential necessity and timing for surgical intervention. We used the ATRS, although not validated, in order to be able to quantify the patient's preoperative symptoms and postoperative outcome.

## Conclusions

Our case contributes to chronic leg tennis management by offering a novel surgical approach. We employed a reverse rectangular flap, taken from the aponeurosis of the musculotendinous junction of the gastrocnemius muscle. We then securely sutured the flap to the muscle belly of the medial gastrocnemius. The sutured area, along with the affected aponeurosis was reinforced by a fascia lata allograft, which we believe also added to the cosmetic result. At 12 months, the patient reported an excellent functional and cosmetic outcome, with an Achilles Tendon Total Rupture Score of 98. This technique adds to the effective treatment protocols that improve outcomes for patients suffering from this condition.
